# Review of EGFR TKIs in Metastatic NSCLC, Including Ongoing Trials

**DOI:** 10.3389/fonc.2014.00244

**Published:** 2014-09-15

**Authors:** Barbara Melosky

**Affiliations:** ^1^Medical Oncology, British Columbia Cancer Agency – Vancouver Centre, Vancouver, BC, Canada

**Keywords:** EGFR TKI, first-line therapy, erlotinib, gefitinib, afatinib, dacomitinib

## Abstract

Recent clinical trials have demonstrated the efficacy of epidermal growth factor receptor (EGFR) tyrosine kinase inhibitors (TKI) in the treatment of patients with advanced metastatic non-small cell lung cancer. Most of these recent trials were conducted in patients with EGFR mutation-positive tumors. As our knowledge of the EGFR mutation and its resistant pathways develops, the complexity of the situation expands. This article briefly reviews the pivotal trials leading to approval of EGFR TKIs in the first-line setting for patients with EGFR mutation-positive non-small cell lung carcinomas. It discusses the historical use of EGFR TKIs after the first-line setting in unselected patients and briefly describes ongoing trials.

## Background

For many years, standard first-line systemic treatment for metastatic NSCLC has consisted of chemotherapy with a two drug combination including a platinum compound and a non-platinum drug such as pemetrexed, gemcitabine, vinorelbine, or a taxane. The typical median time to progression for chemotherapy-treated patients is 4–6 months and median survival is 10–12 months. The advent of epidermal growth factor receptor (EGFR) molecular testing changed the treatment paradigm.

The EGFR or human epidermal growth factor receptor (HER) family contains four members: EGFR (otherwise known as HER1), HER2, HER 3, and HER4. In a normal cell, binding of the epidermal growth factor ligand causes dimerization, phosphorylation, activation of the receptor, and triggering of signaling cascades through pathways such as PI3-Kinase-AKT and RAS/RAF. The presence of an EGFR gene mutation is activating, causing a constant signal to be generated, which leads to cell proliferation and other cancer processes.

Approximately 10–30% of NSCLC patients have an EGFR gene mutation. This mutation is observed at a higher frequency in some subpopulations. In Asian NSCLC cancer patients who never smoked or were only light smokers, this percentage may be as high as 60% (1). For NSCLC patients whose tumors test positive for any EGFR mutations, an oral tyrosine kinase inhibitor (TKI) is now the preferred first-line therapy.

## First-Generation EGFR TKIs

First-generation EGFR TKIs such as erlotinib and gefitinib reversibly compete with adenosine triphosphate (ATP) binding at the tyrosine kinase domain of EGFR. This inhibits ligand-induced EGFR tyrosine phosphorylation, EGFR/HER1 activation, and subsequent activation of the downstream signaling networks (2). Pivotal randomized trials with these first-generation TKIs are chronologically described in the sections below. Although it is tempting to directly compare the results of these studies, a recent publication (3) argues that this type of comparison is invalid due to differences in trial design, comparator choice, and inclusion criteria; readers are urged to refer to Sebastian et al.’s elegant description and critical analysis of these trials (3).

### IDEAL 1 and IDEAL 2 – gefitinib provides a survival advantage in EGFR mutation-unselected patients

The IDEAL 1 (4) and IDEAL 2 (5) phase II trials were two of the first studies to test gefitinib in patients with stage IV NSCLC. These trials demonstrated that both 250 and 500 mg doses of gefitinib were equally active in an EGFR mutation-unselected patient population, resulting in response rates of approximately 20% and median progression-free survival of 2.7 and 2.8 months for the 250 and 500 mg doses of gefitinib, respectively (4). Because both doses showed equivalent results, the lower 250 mg dose was put forward for the registration phase III trials. A subset of patients treated with gefitinib demonstrated a very positive response, but it was unclear why that was the case. At the time, the implications of EGFR mutations were not understood, but we now know that most of these patients likely harbored an EGFR gene mutation.

### NCIC BR.21: erlotinib for an EGFR mutation-unselected patient population improves survival

The NCIC BR.21 phase III trial demonstrated that erlotinib prolonged survival in NCSLC following the failure of first-line or second-line chemotherapy (6). This multicenter, randomized control trial compared erlotinib to placebo in 731 patients with stage IIIB/IV recurrent NSCLC. Study participants who had failed first- or second-line chemotherapy were randomized 2:1 to receive either erlotinib or placebo. One half of the patients had received one prior regimen, and half had received two prior regimens. Patient selection was not based on EGFR mutation status, gender, smoking history, or type of NSCLC.

This study met its primary endpoint of improving overall survival, 6.7 months for erlotinib compared to 4.7 months for placebo (HR 0.70, CI 0.58–0.85, *P* < 0.001). The study demonstrated statistically significant effects in secondary endpoints including progression-free survival of 2.23 months for patients treated with erlotinib compared to 1.84 months for those treated with placebo (HR 0.61, CI 0.51–0.73, *P* < 0.001), time to symptom deterioration, and response rate. Overall, 8.9% of patients achieved an objective response to erlotinib (*P* < 0.001), although mutational analysis was retrospective and only positive in approximately 40 patients. This trial demonstrated a survival benefit in all patients regardless of whether their tumors had an EGFR gene mutation.

Why an EGFR inhibitor was efficacious in the absence of an EGFR mutation is unclear. This reflects the complexity of the EGFR mutation and other downstream signaling pathways, many of which are still to be delineated. As a result of the NCIC BR.21 trial (6), erlotinib was approved and became standard of care in the second or third line setting for patients with NSCLC.

### ISEL: gefitinib provides no survival advantage in an EGFR mutation-unselected population

The Iressa Survival Evaluation in Lung Cancer (ISEL) phase III study was similar to the NCIC BR.21 trial design as it compared an EGFR TKI to placebo in EGFR mutation-unselected NSCLC patients in the second and third line setting (7). Unlike NCIC BR.21, this study failed to meet its endpoint of improved overall survival, with median survival of 5.6 months for patients treated with gefitinib as compared to 5.1 months for patients treated with placebo (HR 0.89, CI 0.77–1.02, *P* = 0.087). There was a pronounced heterogeneity in survival outcomes between groups of patients, most notably those who were never smokers (HR 0.67, CI 0.49–0.92, *P* = 0.012) and those of Asian ancestry (HR 0.66, CI 0.48–0.91, *P* = 0.01). Due to the negative primary results of this trial, gefitinib fell out of use for EGFR mutation-unselected patients in North America.

### Discovery of EGFR mutations

In 2004, two articles were published in prestigious journals by Paez et al. (8) and Lynch et al. (9). Both publications demonstrated that patients who responded well to gefitinib had EGFR gene mutations, and the mutations were located in the region of the gene that encoded the tyrosine kinase domain. Although much discussion centered on whether the presence of the mutation should influence treatment decisions, clarity about the importance of EGFR mutations did not occur until the Iressa Pan Asian Study (IPASS) trial was completed, the mutation status of patients was analyzed, and the biomarker story became clear.

### IPASS trial: gefitinib improves survival in the first line, in an EGFR mutation-enhanced population

The IPASS trial was the study attributed to changing practice. The goal of the IPASS trial was to evaluate the benefit of gefitinib as compared to carboplatin/paclitaxel as first-line treatment for patients with advanced NSCLC (10). Patients selected with this trial had favorable clinical characteristics and included Asian patients with adenocarcinoma, who were non-smokers or former light smokers. Patients treated with gefitinib demonstrated superior progression-free survival as compared to those treated with chemotherapy (HR 0.74, CI 0.65–0.85, *P* < 0.001).

An EGFR biomarker analysis was specified in this protocol, but was retrospective and exploratory. Of 1200 patients, 437 had a tumor specimen that was evaluable for EGFR mutation analysis and of these, 261 patients (59.7%) had tumors that contained EGFR gene mutations. In the subset of EGFR mutation-positive patients, the response rate to gefitinib was 71.2% as compared to 47.3% for carboplatin/paclitaxel. PFS was significantly superior for the EGFR mutation-positive patients treated with gefitinib, 9.5 months as compared to 6.3 months for those treated with chemotherapy (HR 0.48, CI 0.36–0.64, *P* < 0.001). Overall survival was not different, most likely due to crossover; 21.6 months for gefitinib as compared to 21.9 months for carboplatin/paclitaxel.

Iressa Pan Asian Study demonstrated that an EGFR was the most appropriate biomarker for the use of EGFR TKI inhibitors in stage IV non-small cell lung carcinomas and with a significant improvement in PFS and quality of life, gefitinib became standard of care first-line option for NSCLC patients with EGFR-mutated tumor. From this point onward, all TKI trials were conducted in EGFR mutation selected populations and European authorities restricted the use of gefitinib to patients with an EGFR mutation only, regardless of therapeutic line.

### WJOG and NEJSG: Japanese trials testing gefitinib in EGFR mutation selected populations

Two randomized phase III studies compared gefitinib to chemotherapy in the first-line setting (11, 12). Both of these trials, involving NSCLC patients selected on the basis of EGFR mutations, demonstrated a statistically significant increase in progression-free survival for patients treated with gefitinib over chemotherapy. In the West Japan Oncology Group (WJOG) trial, patients treated with gefitinib experienced a median PFS of 9.2 as compared to 6.3 months for those treated with chemotherapy (HR = 0.489, CI 0.336–0.710, *P* < 0.0001) (11). Results were similar in the North-East Japan Study Group (NEJSG), where patients treated with gefitinib experienced a median PFS of 10.8 months compared to 5.4 months for those treated with chemotherapy (HR = 0.30, CI 0.22–0.41, *P* < 0.001) (12). This study was stopped following the results of a planned interim analysis as the gefitinib arm had significantly superior PFS compared to the chemotherapy arm. A high number of patients crossed over to gefitinib (98%); this is the most likely explanation for no difference is overall survival.

### EURTAC trial: erlotinib in the first-line improves progression-free survival

The European Tarceva vs. Chemotherapy (EURTAC) trial was conducted in patients with EGFR mutation positive tumors, and was the first to demonstrate the benefits of an EGFR TKI in a Caucasian population (13). Patients were randomized to receive erlotinib or chemotherapy (cisplatin/gemcitabine or cisplatin/docetaxel) in the first-line setting. Response rate was 58% in the erlotinib arm compared to 15% in the chemotherapy arm (*P* < 0.0001). Progression-free survival was 9.7 months for patients treated with erlotinib and 5.2 months for patients treated with chemotherapy (HR = 0.37, CI 0.25–0.54, *P* < 0.0001) (13). Overall survival was 22.9 months in the erlotinib arm as compared to 18.8 months in the chemotherapy arm (HR = 0.80; *P* = 0.42), most likely confounded by second-line therapy and crossover to erlotinib.

## Second-Generation TKIs

Afatinib and dacomitinib are second-generation EGFR TKIs, and block all HER-family ligands, including HER1 (EGFR), as well as HER2 and HER4. These agents form permanent covalent bonds with the target, irreversibly inhibiting ATP binding at the tyrosine kinase domain. As a result, second-generation TKIs are theoretically more effective in inhibiting EGFR signaling than first-generation erlotinib or gefitinib because the inhibition of EGFR signaling is prolonged for the entire lifespan of the drug-bound receptor molecule (14).

Two phase III trials were conducted to test dacomitinib in EGFR mutation-unselected populations. The Archer 1009 phase III trial compared dacomitinib with erlotinib in EGFR mutation-unselected patients who were previously treated with chemotherapy. The trial did not demonstrate statistically significant improvement in progression-free survival and was discontinued. The NCIC BR.26 trial phase III trial compared dacomitinib with placebo in 736 EGFR mutation-unselected patients with advanced NSCLC previously treated with both chemotherapy and an EGFR TKI. This study also did not meet its objective of prolonging overall survival. Subgroup analysis is currently being conducted in order to understand if there was a difference in response between patients whose tumors harbored an EGFR mutation and those whose tumors did not.

A number of other trials testing the second-generation EGFR TKI dacomitinib are underway and have yet to be published. Archer 1050 is a phase III randomized, open-label trial comparing dacomitinib to gefitinib in a first-line treatment setting in EGFR mutation-positive NSCLC patients. In this trial, approximately 440 patients were randomized 1:1 to dacomitinib or gefitinib. The primary endpoint is PFS by independent review, while the secondary endpoints include PFS by investigator assessment, overall survival, best overall response, duration of response, safety and tolerability, and patient-reported outcomes. As phase II studies of dacomitinib in the first-line treatment setting were promising, we look forward to the results of this phase III study, which will be revealed in mid-2015.

### Afatinib for patients with EGFR mutation positive tumors

LUX-Lung 1 was a phase 2b/3 randomized trial comparing afatinib to best supportive care in unselected patients who had received both a platinum doublet and 3 months of an EGFR TKI, gefitinib, or erlotinib (15). Although progression-free survival was increased, the primary endpoint of overall survival was not. Because of this negative trial, the use of afatinib in patients with an acquired resistance to EGFR TKIs was not approved in any country except Japan.

The pivotal afatinib trial is LUX-Lung 3 (16). This phase III trial randomized 345 patients with NSCLC in the first-line setting who had EGFR mutation-positive tumors to receive either afatinib or cisplatin/pemetrexed. For this study, all EGFR mutations from codons 18–21 were analyzed. While the majority of patient tumors harbored common EGFR mutations (Del-19 and Point 21 L858R), approximately 10% of patients had uncommon EGFR mutations. The primary endpoint of this trial was progression-free survival and secondary endpoints included overall survival, objective response rate, and quality of life.

Afatinib treatment led to an increase in the objective response rate compared with chemotherapy treatment (56.1 vs. 22.6%). Patients randomized to afatinib experienced a significant improvement in median progression-free survival compared with those randomized to chemotherapy, 11.1 vs. 6.9 months, respectively (HR 0.58, CI 0.43–0.78, *P* = 0.0004). The treatment effect of afatinib was more pronounced when comparing progression-free survival in the pre-defined subgroup of patients with the common Del-19 or Point 21 L858R EGFR mutations. In this subgroup, patients treated with afatinib experienced progression-free survival of 13.6 months as compared to 6.9 months for those treated with chemotherapy (HR 0.47, CI 0.34–0.65, *P* < 0.0001) (16).

The LUX-Lung 6 trial, conducted in Asia, confirmed the value of afatinib in the population of patients with EGFR mutation-positive tumors (17). This phase III, open-label trial randomized 364 NSCLC patients in a 2:1 fashion to receive afatinib or gemcitabine/cisplatin. The primary endpoint in this study was progression-free survival and secondary endpoints included objective response rate, disease control rate, patient-reported outcomes, and safety.

A statistically significant improvement in progression-free survival was demonstrated between patients treated with afatinib as compared to those treated with chemotherapy, 11.0 vs. 5.6 months, respectively (HR 0.28, CI 0.20–0.39, *P* < 0.0001) (17). The progression-free survival benefit was consistent across all subgroups, including all mutation categories. The percentage of LUX-Lung 6 patients with a confirmed objective response was 67% in the afatinib group as compared to 23% in the chemotherapy group. Overall, the results of the LUX-Lung 6 trial support the efficacy observations (progression-free survival and objective response rate) demonstrated in the LUX-Lung 3 trial.

To date, none of the published randomized EGFR TKI trials have demonstrated a statistically significant improvement in overall survival. In the American Society of Clinical Oncology meeting in Chicago 2014, a pooled analysis of LUX-Lung 3 and LUX-Lung 6 was presented (18). Although the pooling of clinical trial results in this way is controversial, the results are interesting. According to this analysis, the overall survival of LUX-Lung 3 was 31.6 months for patients treated with afatinib as compared to 28.2 months for those treated with chemotherapy (pemetrexed/cisplatin) (HR: 0.78). The pooled overall survival analysis of LUX-Lung 6 showed that patients treated with afatinib had a median survival of 23.6 months as compared to 23.5 months when treated with gemcitabine/cisplatin (HR 0.8). Although both hazard ratios are approximately 0.8, neither of the *P* values were significant.

The pooled analysis showed an important improvement in overall survival in patients whose tumors had the most common EGFR mutations, Del-19 and Point 21 L858R. In the sub-population of patients with these mutations, the median overall survival in the afatinib arm was 27.3 months, which was significantly improved over median overall survival of 24.3 months in the chemotherapy arm (HR 0.81, *p* = 0.037).

The most interesting analysis concerned the subpopulation of patients whose tumors had harbored the Del-19 deletion, where a significant improvement in overall survival was seen in both LUX-Lung 3 and LUX-Lung 6 trials. In the LUX-Lung 3 trail, the median survival was 33.2 months for Del-19 patients treated with afatinib as compared to 21.1 months with chemotherapy (pemetrexed/cisplatin) (HR 0.54). In LUX-Lung 6, the median survival was 31.4 months for Del-19 patients treated with afatinib as compared to 18.4 months for patients treated with chemotherapy (gemcitabine/cisplatin) (HR 0.64). The author concluded that the patients with Del-19 and Point 21 L858R mutations may constitute very different populations, and may require different treatment strategies (18).

A highly anticipated trial is LUX-Lung 7. This phase III, open-label trial randomized 316 patients with EGFR mutation-positive advanced adenocarcinoma to receive either afatinib or gefitinib. The primary endpoint for the trial, which completed in July 2013, was overall survival. We await the results eagerly.

Clinical trials with the third-generation EGFR TKIs are underway. These inhibitors work to selectively inhibit tumors that harbor the acquired T790 mutation.

Currently, there are more than 350 open trials for EGFRs in NSCLC, and at least 20 of these are phase III. Indeed, this is a very exciting time in the evolution of our knowledge of the EGFR TKI inhibitors, and we expect outstanding advances in the care of our patients with non-small cell lung carcinoma.

## Conflict of Interest Statement

The author declares that the research was conducted in the absence of any commercial or financial relationships that could be construed as a potential conflict of interest. The Guest Associate Editor Vera Hirsh declares that, despite having collaborated with author Barbara Melosky, the review process was handled objectively and no conflict of interest exists.

## Abbreviations

ATP, adenosine triphosphate; CI, confidence interval; EGFR, epidermal growth factor receptor; HER, human epidermal growth factor receptor; HRQoL, health-related quality of life; NCI, National Cancer Institute; NSCLC, non-small cell lung cancer; ORR, objective response rate; OS, overall survival; PFS, progression-free survival; RCT, randomized clinical trial; TKI, tyrosine kinase inhibitor.
